# Akt Deficiency Attenuates Muscle Size and Function but Not the Response to ActRIIB Inhibition

**DOI:** 10.1371/journal.pone.0012707

**Published:** 2010-09-15

**Authors:** Marcus D. Goncalves, Emidio E. Pistilli, Anthony Balduzzi, Morris J. Birnbaum, Jennifer Lachey, Tejvir S. Khurana, Rexford S. Ahima

**Affiliations:** 1 Department of Medicine, University of Pennsylvania School of Medicine, Philadelphia, Pennsylvania, United States of America; 2 Department of Physiology and Pennsylvania Muscle Institute, University of Pennsylvania School of Medicine, Philadelphia, Pennsylvania, United States of America; 3 Institute for Diabetes, Obesity and Metabolism, University of Pennsylvania School of Medicine, Philadelphia, Pennsylvania, United States of America; 4 Acceleron Pharma, Cambridge, Massachusetts, United States of America; McMaster University, Canada

## Abstract

**Background:**

Akt is a critical mediator of developmental skeletal muscle growth. Treatment with a soluble ActRIIB fusion protein (ActRIIB-mFc) increases skeletal muscle mass and strength by inhibiting myostatin and related peptides. Recent *in vitro* studies have suggested that Akt signaling is necessary for the ability of ActRIIB inhibition to induce muscle hypertrophy. Thus, we hypothesized that mice deficient in either Akt1 or Akt2 would not respond to *in vivo* inhibition of ActRIIB with ActRIIB-mFc treatment.

**Methodology and Principal Findings:**

We analyzed body composition and muscle parameters in wild-type C57BL/6J and Akt1 and Akt2 knockout mice, and compared the responses to blockade of ActRIIB signaling via ActRIIB-mFc treatment. Mice lacking Akt1 or Akt2 had reduced muscle mass, grip strength and contractile force. However, deficiency of Akt1 or Akt2 did not prevent the ability of ActRIIB-mFc treatment to induce muscle hypertrophy, or increase grip strength and contractile force. Akt1 and Akt2 deficient mice responded similarly as wild type mice to ActRIIB-mFc treatment by increasing fiber size.

**Conclusions and Significance:**

Akt1 and Akt2 are important for the regulation of skeletal muscle mass and function. However, these Akt isoforms are not essential for the ability of ActRIIB inhibition to regulate muscle size, fiber type, strength or contractile force.

## Introduction

Skeletal muscle adapts to environmental stimuli and alters its mass accordingly. New myofilaments are added to existing myofibrils through an increase in protein synthesis under the control of mechanical load, nutrients and hormones. Signaling through the phosphatidylinositol 3-kinase (PI3K)/Akt pathway increases protein synthesis [1], [2]. Akt is sufficient to induce muscle hypertrophy as demonstrated by muscle-specific expression of a constitutively active form of Akt [3]. Three Akt isoforms (Akt1/2/3) have been identified in mice and humans, and implicated in the regulation of growth and metabolism [4], [5]. Germline ablation of Akt1 results in a proportional reduction in body size [6], [7], [8]. In contrast, conditional Akt1 overexpression in muscle resulted in muscle hypertrophy and increase in strength [9], [10]. Disruption of Akt2 in mice resulted in insulin resistance, hyperglycemia, hyperinsulinemia, and glucose intolerance [11], [12]. As with Akt1, mice lacking Akt2 develop a mild growth deficiency [11], [12]. Deficiency of both Akt1 and Akt2 resulted in multiple developmental defects including muscle atrophy [13].

Another key mediator of muscle size is the activin receptor type IIB (ActRIIB) [14]. ActRIIB is a type II transforming growth factor (TGF)-β superfamily receptor that is emerging as a key player in the regulation of muscle size and strength [15], [16]. Ligands, including myostatin and GDF-11, bind to the ActRIIB leading to phosphorylation and nuclear translocation of Smad2/3, which mediates muscle atrophy [17]. Inhibition of ActRIIB signaling can be achieved by genetically overexpressing regulatory proteins (such as follistatin), which binds and inhibits endogenous TGFβ superfamily ligands [18], [19]. Other methods for inhibiting ActRIIB include expression of a dominant-negative form of ActRIIB [19] or postnatal injection of a decoy ActRIIB receptor [15], [16]. These methods result in a dramatic increase in muscle mass, more than what is seen in myostatin deficiency alone, indicating that more than one ActRIIB ligand is important for the control of muscle size [16], [19], [20].

Studies done in cell lines and through electroporation have demonstrated a dominant role of Akt signaling in the regulation of myocellular hypertrophy resulting from inhibition of ActRIIB [10], [21], [22], [23], [24], [25]. It is unknown, however, whether the interaction between Akt and ActRIIB signaling is responsible for the resulting hypertrophy *in vivo*. We have previously shown that treatment with a decoy ActRIIB soluble receptor (ActRIIB-mFc) increased muscle size and contraction force [15], [26]. We hypothesized that deficiency of Akt1 or Akt2 would prevent the *in vivo* effects of ActRIIB inhibition on muscle.

## Results

### Effects of Akt deficiency and ActRIIB blockade on body composition

**Figure 1:**
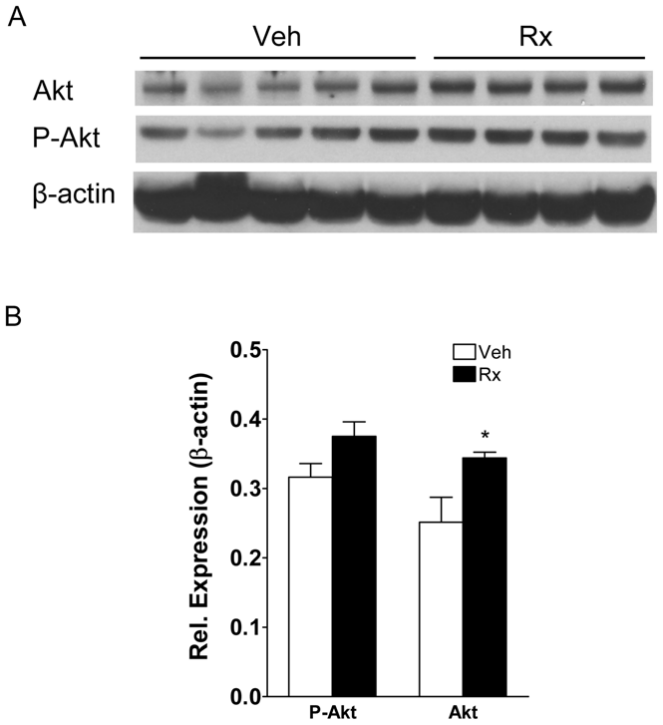
Effects of ActRIIB inhibition on Akt levels in gastrocnemius muscle. (A) Phosphorylated Akt (Ser473), total Akt1/2/3, and β-actin levels after 10 weeks of vehicle- (Veh) or ActRIIB-mFc treatment. (B) Total and p-Akt levels relative to β-actin. *p<0.05 vs. vehicle.

We first performed immunoblotting on lysates from gastrocnemius muscles to assess the effects of ActRIIB-mFc on ActRIIB signaling on the levels of total Akt and phospho-Akt (Ser473). ActRIIB-mFc treatment increased total Akt significantly (Fig. 1). The level of phospho-Akt increased slightly in response to ActRIIB-mFc, but this was not significant (Figure 1).

**Figure 2:**
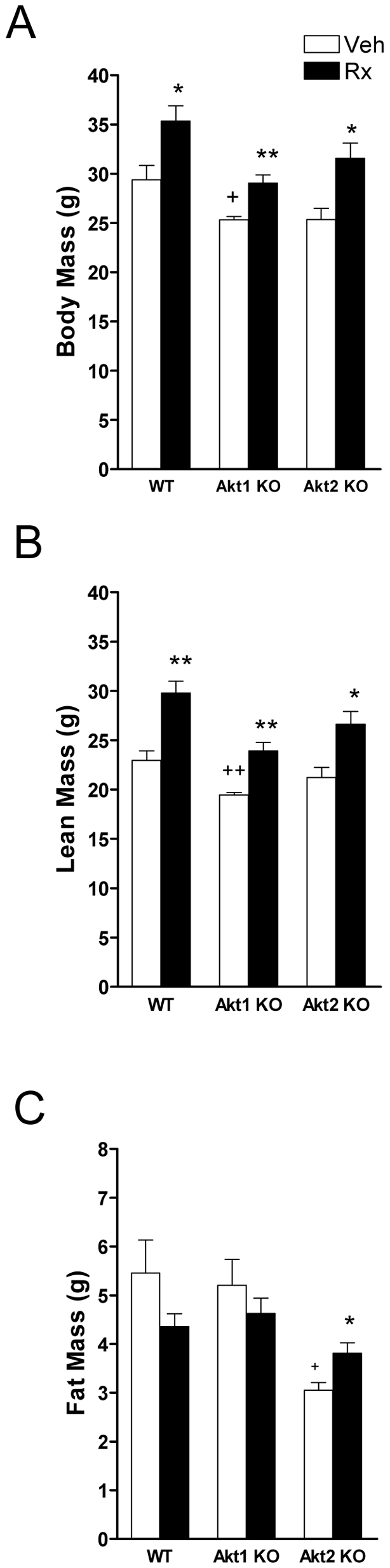
Effects of Akt deficiency and ActRIIB inhibition on body composition. Effects of genotype and ActRIIB-mFc treatment (Rx, black bar) or vehicle (Veh, white bar) in wild-type (WT), Akt1 knockout mice (Akt1 KO), and Akt2 knockout mice (Akt2 KO) on (A) body weight, (B) percentage lean, (C) percentage fat. Data are mean +/− SEM, n = 5. + *P*<0.05 vs. WT treated with vehicle; ++ *P*<0.01 vs. WT treated with vehicle; *P<0.05 vs. same genotype treated with vehicle; **P<0.01 vs. same genotype treated vehicle.

We compared body composition among wild type (WT) and Akt1 knockout (ko) and Akt2ko mice, and also examined the effects of ActRIIB-mFc after 10 weeks. There was a significant effect on body weight with respect to genotype (Figure 2A, p = 0.0008). Akt1ko mice weighed significantly less than WT (14.1%, p = 0.0265) and Akt2ko mice tended to weigh less than WT (13.8%, p = 0.0751). There was a significant effect on lean mass (Figure 2B, p<0.01), and fat mass (Figure 2C, p<0.01) with respect to genotype. Lean mass was 15% lower in Akt1ko mice as compared to WT (Figure 2B, p<0.01) whereas Akt2ko mice were not significantly different. Fat mass was 44% decreased in Akt2ko mice as compared to WT mice (Figure 2C, p<0.05), whereas it was unchanged in Akt1ko mice. The proportions of lean and fat tissue were unchanged from WT in Akt1ko mice (WT lean: 78.3±1.7%, WT fat: 18.4±1.6%; Akt1ko lean: 76.9±1.5%, Akt1ko fat: 20.5±1.9%). In contrast, Akt2ko mice had a significant increase in percentage lean tissue and a decrease in percentage fat (Akt2ko lean: 83.7±0.3%, fat: 12.1±0.4%, p<0.01 vs. WT for both) when compared to WT.

ActRIIB-mFc treatment had a significant effect on body weight (120% in WT, 115% in Akt1ko, and 124% in Akt2ko. Figure 2A, p<0.0001). In accordance with changes in body weight, lean mass was significantly affected by treatment (Figure 2B, p<0.0001). Fat mass tended to decrease with ActRIIB-mFc treatment in WT and Akt1ko mice cohorts but increased in Akt2ko mice (Figure 2C, p<0.05). With ActRIIB-mFc treatment, the proportions of lean tissue significantly increased in WT and Akt1ko mice (WT: 84.3±0.3%; Akt1ko 82.3±0.7%, p<0.01 for both) while the proportion of fat tissue decreased (WT: 12.3±0.4%; Akt1ko 16.0±1.2%, p<0.05 for both). In contrast, Akt2ko mice had no change in body composition (Akt2ko lean: 84.4±0.7%, fat: 12.1±0.4%) with treatment.

### Effects of Akt deficiency and ActRIIB blockade on muscle structure

**Figure 3:**
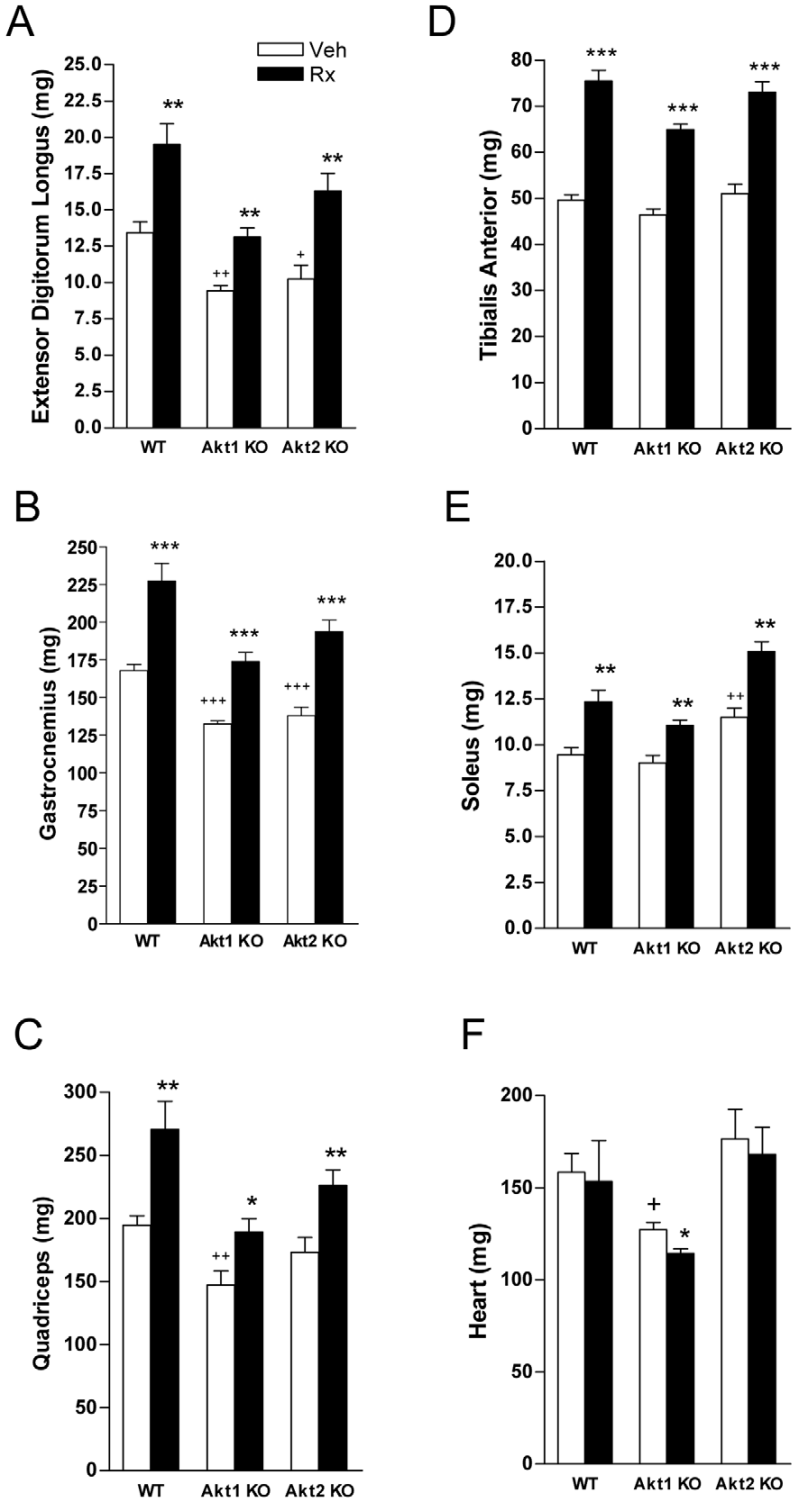
Effects of Akt deficiency and ActRIIB inhibition on skeletal muscle weights. Effects of genotype and ActRIIB-mFc treatment (Rx, black bar) or vehicle (Veh, white bar) in wild-type (WT), Akt1 knockout mice (Akt1 KO), and Akt2 knockout mice (Akt2 KO) on (A) Extensor digitorum longus, (B) Gastrocnemius (C) Quadriceps, (D) Tibialis Anterior, (E) Soleus, and (F) Heart. Data are mean +/− SEM, n = 5; + *P*<0.05 vs. WT treated with vehicle; ++ *P*<0.01 vs. WT treated with vehicle; +++*P*<0.0001 vs. WT treated with vehicle. *P<0.05 vs. same genotype treated with vehicle **P<0.01 vs. same genotype treated with vehicle; ***P<0.0001 vs. same genotype treated with vehicle.

We examined the effects of Akt deficiency on various skeletal muscles (Figures 3A–E). The weights of extensor digitorum longus (EDL), gastrocnemius, anterior quadriceps, tibialis anterior, and soleus were significantly affected by genotype (p<0.01 for all). The mass of EDL, gastrocnemius, and anterior quadriceps was significantly decreased in Akt1ko compared to WT (Figure 3A–C). Akt2ko mice showed a decrease in EDL and gastrocnemius mass (Figure 3A–B) and a 21% increase in soleus mass as compared to WT (Figure 3E, p<0.01). Heart mass was significantly affected by genotype (Figure 3F, p = 0.0012). Akt1ko heart mass was 20% lower than WT (p<0.05) but unaffected in Akt2ko. ActRIIB-mFc treatment increased the weights of all skeletal muscles measured (Figures 3A–E). Heart mass was decreased in Akt1ko mice with treatment however not in WT or Akt2ko mice (Figure 3F, p<0.05).

**Figure 4:**
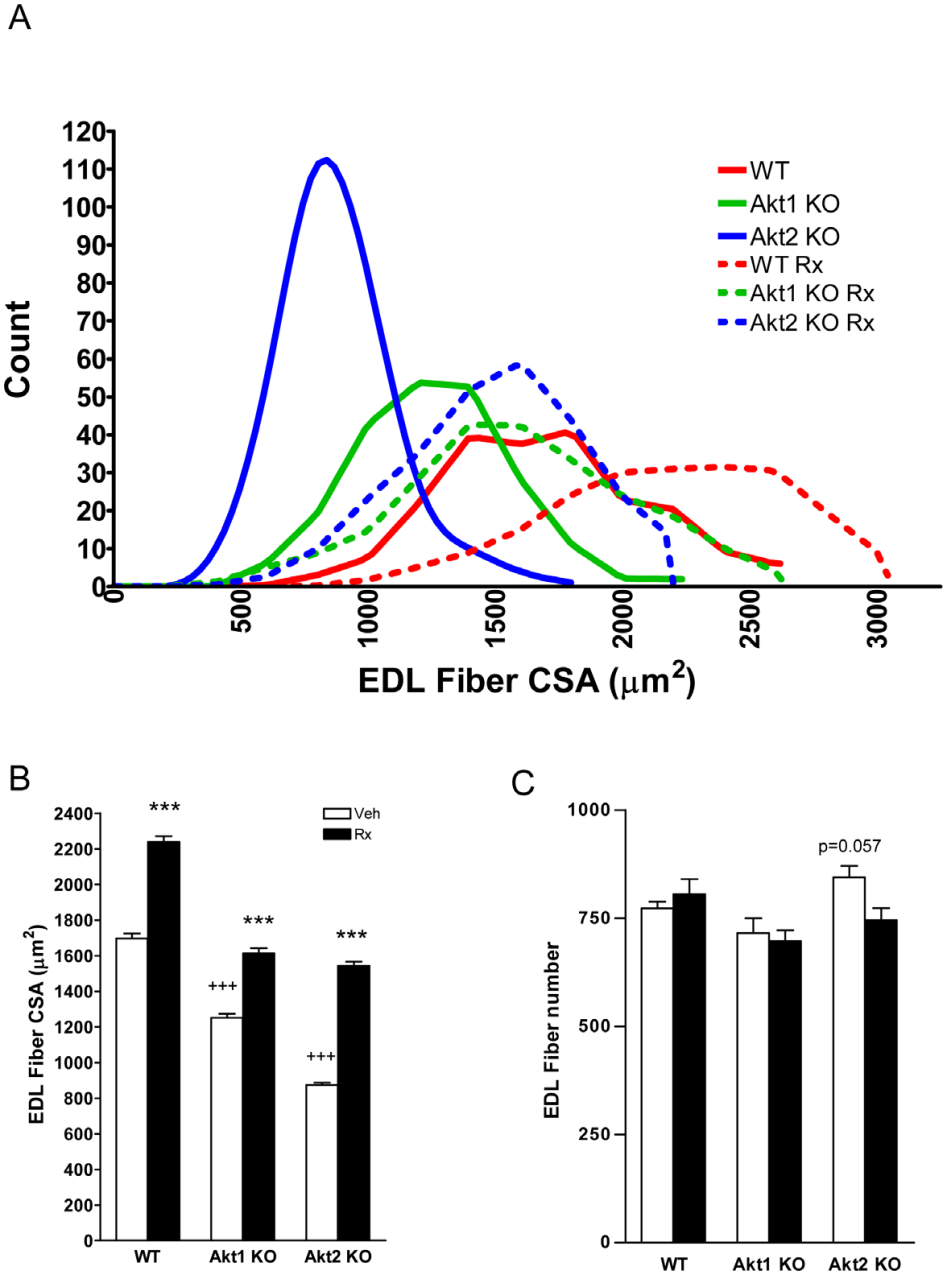
Effects of Akt deficiency and ActRIIB inhibition on EDL fiber size and distribution. (A) Effects of genotype and ActRIIB-mFc treatment on EDL fiber cross-sectional area distributions. Wild-type (WT, red), Akt1 knockout mice (Akt1 KO, green), Akt2 knockout mice (Akt2 KO, blue), ActRIIB-mFc -treated WT (WT Rx, red-dashed), ActRIIB-mFc -treated Akt1 KO (Akt1 KO Rx, green dashed), and ActRIIB-mFc -treated Akt2 KO (Akt2 KO Rx, blue dashed) EDL fiber distributions; n≥792 fibers per group; (B) Single fiber cross sectional areas per genotype (Veh, white bar) and treatment (Rx, black bar); (C) Number of fibers in the EDL. Data are mean +/− SEM, n = 5; +++*P*<0.0001 vs. WT treated with vehicle; ***P<0.0001 vs. same genotype treated with vehicle.

Next, we compared the effects of Akt deficiency and ActRIIB blockade on EDL and soleus muscles as examples of glycolytic versus oxidative muscles, respectively. The mean cross sectional area (CSA) of EDL was significantly altered with genotype (WT: 1.75±0.14 mm^2^, Akt1ko: 1.22±0.07 mm^2^, Akt2ko: 1.25±0.13 mm^2^, p<0.0001). There was a shift to the left in the distribution of EDL fiber CSA in Akt1ko and Akt2ko mice (Figure 4A) as represented by a decrease in mean EDL fiber CSA (Figure 4B, p<0.0001 for both Akt1ko and Akt2ko vs. WT). The number of EDL fibers was affected by genotype (Figure 4C, p<0.01) however direct comparisons to WT were non-significant. ActRIIB-mFc treatment increased the mean EDL fiber CSA and resulted in a right shift in the distribution of fibers in WT, Akt1ko and Akt2ko mice (Figure 4A). There was no significant change in the total number of EDL muscle fibers following ActRIIB-mFc treatment (Figure 4C). These data confirmed that ActRIIB-mFc induced EDL muscle hypertrophy and not hyperplasia [15].

**Figure 5:**
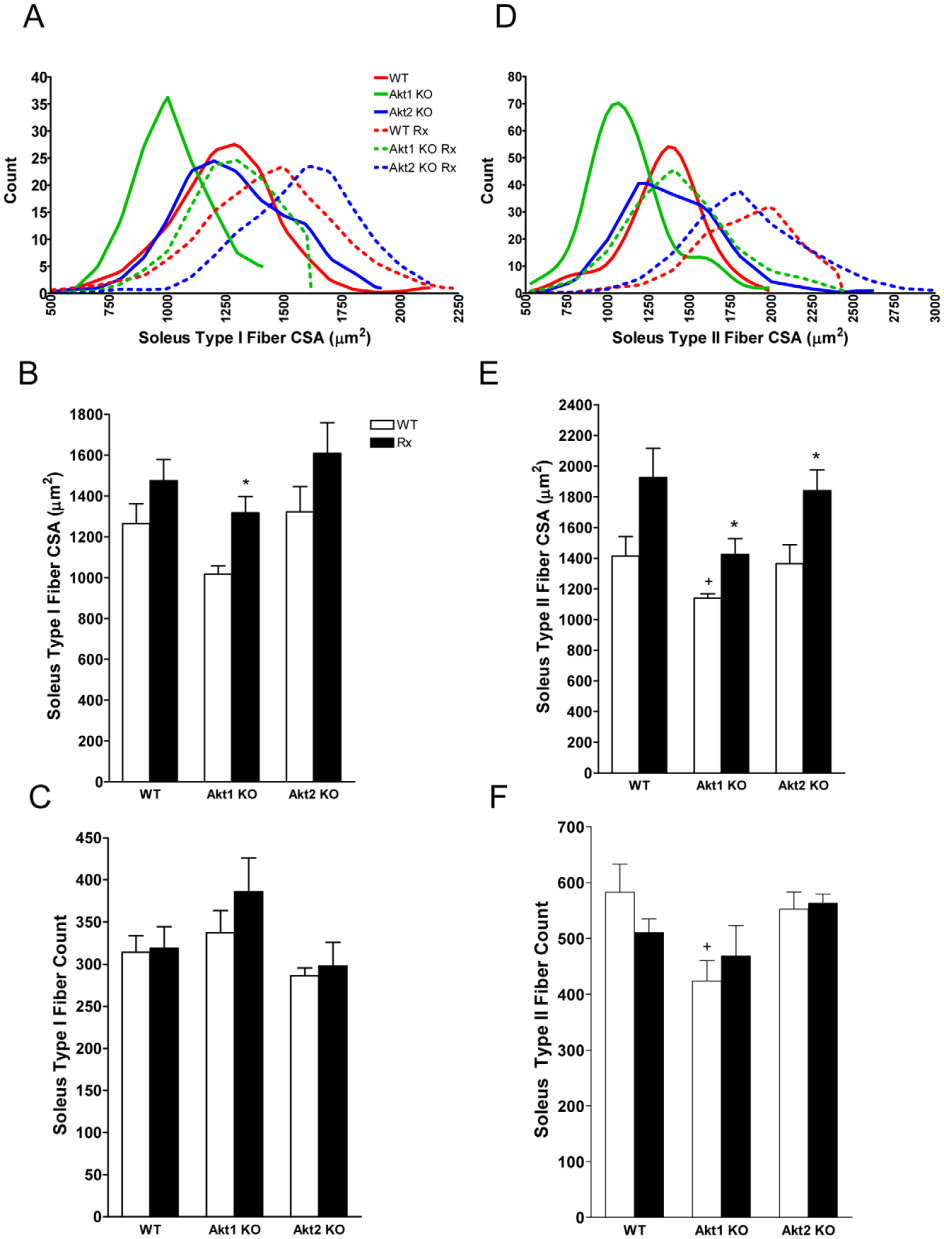
Effects of Akt deficiency and ActRIIB inhibition on soleus fiber size and distribution. (A) Effects of genotype and ActRIIB-mFc treatment on soleus type I and type II fiber cross-sectional area distributions. Wild-type (WT, red), Akt1 knockout mice (Akt1 KO, green), Akt2 knockout mice (Akt2 KO, blue), ActRIIB-mFc -treated WT (WT Rx, red-dashed), ActRIIB-mFc -treated Akt1 KO (Akt1 KO Rx, green dashed), and ActRIIB-mFc -treated Akt2 KO (Akt2 KO Rx, blue dashed) on soleus type I fiber distribution; n≥393 fibers per group. (B) Type I single fiber cross sectional area (CSA) per genotype (Veh, white bar) and treatment (Rx, black bar); (C) Number of type I fibers in the soleus; (D) Soleus type II fiber distribution; n≥458 fibers per group. (E) Type II single fiber CSA per genotype and treatment; (F) Number of type II fibers in the soleus; Data are mean +/− SEM, n = 5; +*P*<0.05 vs. WT treated with vehicle; *P<0.05 vs. same genotype treated with vehicle.

In contrast to EDL, Akt deficiency did not change the soleus CSA (WT: 1.64±0.27, Akt1ko: 1.48±0.10, Akt2ko: 1.56±0.04 mm^2^) or total number of fibers (WT: 896.5±67.5, Akt1ko: 761.0±58.7, Akt2ko: 838.8±37.6). The proportion of type II fibers was reduced in Akt1ko but not in Akt2ko (WT: 64.8±1.2%, Akt1ko: 55.6±1.4%, Akt2ko: 65.8±0.9%; WT vs. Akt1ko p<0.05). Type I fiber CSA tended to be smaller in soleus muscle of Akt1ko mice as compared to WT (Figure 5A–B, p = 0.0582), but the number of type I fibers was not significantly affected (Figures 5C). Type II fibers were smaller in the soleus muscle of Akt1ko mice (Figure 5D–E, p<0.05) and the number of type II fibers was reduced (Figures 5F, p<0.05). ActRIIB-mFc increased soleus CSA (WT: 1.88±0.18, *Akt1*: 1.75±0.15, *Akt2*: 1.94±0.17 mm^2^, p<0.05) but did not change the total number of fibers (WT: 828.8±29.2, *Akt1*: 854.3±74.7, *Akt2*: 860.5±42.2). ActRIIB-mFc did not alter the fiber composition of soleus muscles in WT, Akt1ko and Akt2ko mice (Percent Type II in WT: 61.5±2.4%, Akt1ko: 54.7±3.3%, Akt2ko: 65.6±1.7%). The mean CSA of type I and type II fibers was significantly altered with ActRIIB-mFc treatment (Figure 5B, 5E p<0.01 for both) and the distribution shifted to the right in all genotypes following ActRIIB-mFc treatment (Figure 5A, 5D). However, the total numbers of type I and type II fibers were not affected by ActRIIB-mFc (Figure 5C, 5F).

### Effects of Akt deficiency and ActRIIB blockade on muscle function

**Figure 6:**
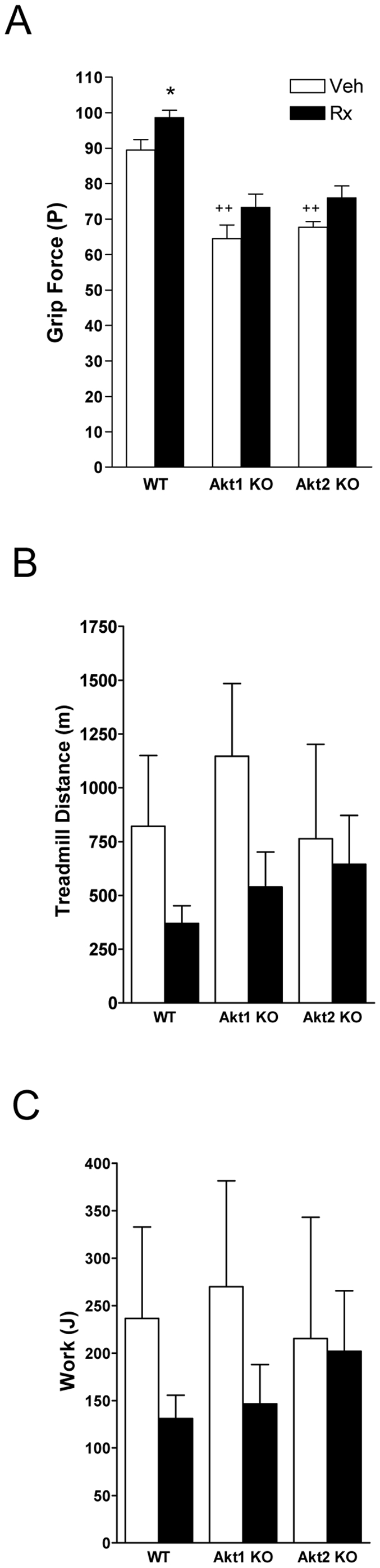
Effects of Akt deficiency and ActRIIB inhibition on *in vivo* muscle function. Effects of genotype (Veh, white bar) and ActRIIB-mFc treatment (Rx, black bar) in wild-type (WT), Akt1 knockout mice (Akt1 KO), and Akt2 knockout mice (Akt2 KO) on (A) forelimb grip strength; (B) distance ran during an endurance exercise protocol; (C) work done during an endurance protocol. Data is mean +/− SEM, n = 5; + *P*<0.05 vs. WT treated with vehicle; ++ *P*<0.01 vs. WT treated with vehicle. *P<0.05 vs. same genotype treated with vehicle.

Forelimb grip strength was reduced in Akt1ko and Akt2ko compared to WT (Figure 6A, p<0.01). We subjected WT, Akt1ko and Akt2ko mice to an endurance treadmill exercise test until fatigue. There were no apparent differences in the distance run (Figure 6B), or work done during exercise among the genotypes (Figure 6C). ActRIIB-mFc increased grip strength across genotypes (Figure 6A, p = 0.0024). Despite a clear trend we observed a non-significant decrease in distance run (Figure 6B) and work done (Figure 6C) during endurance testing in WT and Akt1ko mice with treatment.

**Figure 7:**
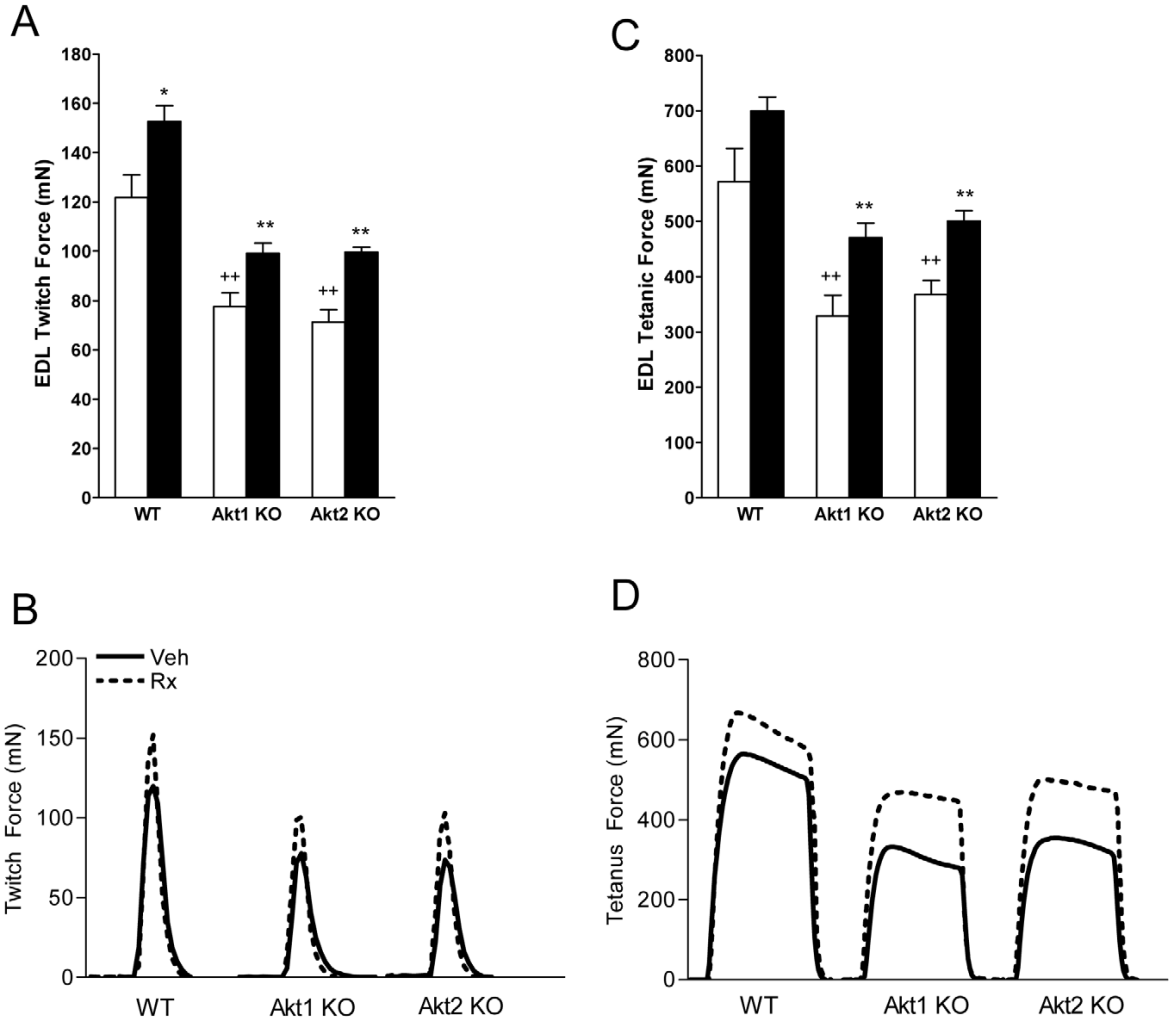
Effects of Akt deficiency and ActRIIB inhibition on EDL contraction. Effects of genotype (Veh, white bar) and ActRIIB-mFc treatment (Rx, black bar) on EDL *ex vivo* force generation. (A) Peak twitch force (mN) in wild-type (WT), Akt1 knockout mice (Akt1 KO), and Akt2 knockout mice (Akt2 KO); (B) Representative twitch recordings; (C) Peak tetanic force (mN). (D) Representative tetanic recordings. Data in (A, C) are mean +/− SEM, n = 5; + *P*<0.05 vs. WT treated with vehicle; ++ *P*<0.01 vs. WT treated with vehicle. *P<0.05 vs. same genotype treated with vehicle **P<0.01 vs. same genotype treated with vehicle.

Muscle contraction was measured in EDL muscles *ex vivo*. Maximum twitch force was decreased in Akt1ko and Akt2ko as compared to WT (Figure 7A, p<0.01 for both). However, there was no significant difference in the twitch dynamics among WT, Akt1ko or Akt2ko EDL muscles (Figure 7B). The time to peak contraction (CT) was similar across genotypes (WT: 35.0±3.3, Akt1ko: 32.0±2.5, Akt2ko: 28.8±2.3 msec). The time to half maximum force (½RT) was not affected by genotype (WT: 32.5±1.6, Akt1ko: 31.0±2.3, Akt2ko: 27.5±1.6 msec). The maximum tetanic force was decreased in Akt1ko and Akt2ko (Figure 7C, p<0.01), but tetanus dynamics were not different (Figure 7D).

ActRIIB-mFc increased the EDL twitch force across genotypes (Figure 7A, p<0.0001). CT was not affected by ActRIIB-mFc (WT: 33.8±1.8, Akt1ko: 32.0±2.5, Akt2ko: 27.5±1.6 msec). In contrast, ½RT was decreased by ActRIIB-mFc across genotypes (WT: 26.3±1.8, *Akt1*: 26.0±1.6, *Akt2*: 25.0±1.9 msec, p<0.01), while the maximum tetanic force was increased by ActRIIB-mFc treatment (Figure 7C, p<0.0001).

### Effect of Akt deficiency and ActRIIB blockade on expression of atrophy-related genes

We detected no significant effect of Akt1 or Akt2 deficiency on the expression of atrophy-related E3-ubiquitin ligases, MAFbx/atrogin-1 and MuRF1 (Figure S1). Inhibition of ActRIIB signaling resulted in non-significant decrease in expression of MAFbx and MuRF1 in WT mice, and significant decrease in Akt2ko mice (Figure S1, p<0.01 and p<0.05 respectively).

### Effects of Akt deficiency and ActRIIB blockade on Akt signaling

**Figure 8:**
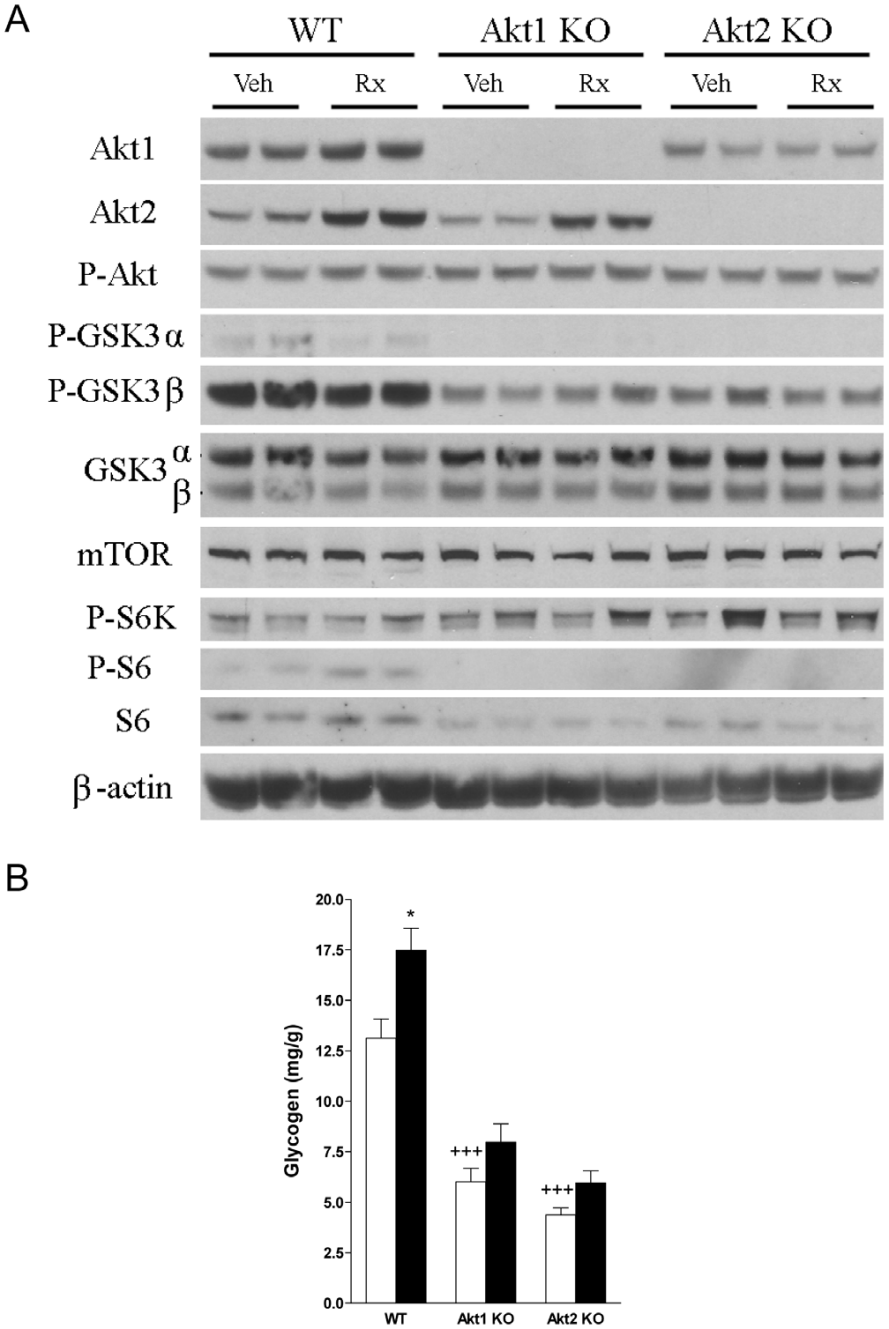
Effects of ActRIIB inhibition on Akt signaling and glycogen content. (A) Immunoblots of vehicle (Veh) and ActRIIB-mFc treated (Rx) wild-type (WT), Akt1 knockout (Akt1ko), and Akt2 knockout (Akt2ko) gastrocnemius lysates. P: phospho-specific antibody (B) Effects of genotype (Veh, white bar) and ActRIIB-mFc treatment (Rx, black bar) on quadriceps glycogen content (mg glycogen per g muscle). +++*P*<0.0001 vs. WT Veh, *p<0.05 vs. vehicle.

WT, Akt1ko, and Akt2ko gastrocnemius lysates were blotted for expression of Akt signaling proteins (Figure 8A). Total Akt1 and Akt2 levels were lower in Akt1ko and Akt2ko lysates compared to WT whereas phospho-Akt levels were similar across genotypes. Total glycogen synthase kinase-3 (GSK-3) levels in Akt mutant lysates were comparable to WT, but phosphorylated GSK-3 levels were reduced in Akt1ko and Akt2ko. Mammalian target of rapamycin (mTOR) levels were similar across genotypes, while phosphorylated S6 kinase (S6K) tended to be variable among WT, Akt1ko and Akt2ko (Figure 8A). Levels of phosphorylated S6 ribosomal protein were undetectable in both Akt1ko and Akt2ko, and total S6 levels were decreased. Glycogen content was significantly lower in Akt1ko and Akt2ko compared to WT (Figure 8B, p<0.0001 in Akt1ko and Akt2ko versus WT). ActRIIB inhibition increased Akt1 and Akt2 in WT, and Akt2 in Akt1ko mice. We did not detect changes in phosphorylated Akt, phosphorylated GSK-3, total GSK-3, mTOR, and phosphorylated S6K in response to ActRIIB inhibition. Levels of phosphorylated S6 were increased in response to ActRIIB inhibition in WT, whereas total S6 levels were not altered substantially. Glycogen content was significantly increased by ActRIIB inhibition in WT (p<0.05), and tended to increase in Akt1ko and Akt2ko mice (Figure 8B).

## Discussion

Akt plays a pivotal role in mediating the responses to insulin and growth factors [1], [27]. Akt1 and Akt2 are highly expressed in insulin-sensitive tissues. Constitutive expression of Akt1 in L6 myoblasts promotes glucose and amino acid uptake and protein synthesis [28]. Overexpression of Akt2, but not Akt1, stimulates C2C12 muscle cell differentiation, suggesting that Akt1 and Akt2 isoforms are necessary for optimal muscle growth [29]. Mice lacking Akt1 are smaller and have been reported to show a decrease in muscle size compared with wild-type mice [6], [7], [8]. Akt2 deficient mice exhibit a diabetic phenotype, associated with reduced glucose uptake in the EDL but not the soleus muscle, suggesting a fiber type specific insulin resistance (15). Mice with Akt1/Akt2 deficiency develop multiple developmental defects and severe muscle atrophy, indicating that both Akt isoforms contribute to the determination of muscle size. However, detailed analyses of the effects of Akt1 and Akt2 deficiency on body composition, muscle structure and function are lacking.

Our studies revealed differential effects of Akt1 and Akt2 deficiency on body composition and skeletal muscle. In agreement with previous reports, Akt1ko mice were smaller, and displayed proportional decreases in body and lean mass compared to wild-type mice [6], [7], [8]. In contrast, Akt2ko mice weighed less than wild-type mice, an effect explained mainly by a decrease in body fat. Akt1ko mice had significant decreases in the weights of gastrocnemius, EDL, and quadriceps, i.e. muscles composed mainly of type II fibers [30]. By examining the histology of EDL, we found the Akt1 deficiency was associated with a significant decrease in the cross-sectional area of fibers. In contrast to EDL, Akt1 deficiency did not change the weight of soleus muscle, which is composed of both type I and type II fibers. Nonetheless, Akt1 deficiency decreased the number of type II fibers in soleus muscle. Thus, Akt1 is essential for the determination of the size and number of type II fibers. Our results are in agreement with Easton et al. who demonstrated that cardiomyocytes from Akt1ko mice have smaller CSA [8]. Akt2 deficiency resulted in significant reductions the weights of EDL and gastrocnemius, whereas the weight of soleus muscle was increased relative to wild-type mice. The reduction in EDL weight in Akt2ko was explained, at least partly, by a reduction in fiber cross-sectional area. The reasons for the increase in soleus muscle weight in Akt2ko mice are unknown. There were no apparent changes in the fiber cross-sectional area or number of fibers counted at the mid-section of the soleus muscle. However, it is possible that an increase in soleus muscle triglyceride or extracellular matrix that were not measured in our study may have played a role in the increase in soleus weight observed in Akt2ko mice.

Overexpression of constitutively active Akt1 or Akt2 in muscle results in increased levels of phosphorylated S6K and glycogen content [31]. In agreement with these findings, we found that Akt1 and Akt2 deficient animals have decreased phosphorylated S6 levels, a marker of S6K activity, and decreased levels of phosphorylated GSK-3 and glycogen content.

While Akt is important for the development of muscle size and function, we found that the hypertrophy resulting from ActRIIB inhibition with ActRIIB-mFc was not dependent on either Akt1 or Akt2 isoforms. Similar to wild-type mice, ActRIIB-mFc treatment increased the mean EDL fiber cross-sectional area and shifted the fiber distribution to the right in Akt1ko and Akt2ko mice, consistent with type II fiber hypertrophy [15], [32], [33]. ActRIIB-mFc treatment induced type I and type II fiber hypertrophy in the soleus muscle. This result is in agreement with a recent study in which ACE031 (the humanized analogue of ActRIIB-mFc) treatment increased the mean fiber cross-sectional area in plantaris, a predominantly type II muscle, and as well as type I and type II fibers in the soleus muscle [26].

Our results suggest that the increase in muscle mass resulting from ActRIIB-mFc treatment is specialized for strength. Forelimb grip strength and EDL twitch and tetanic contractions were increased by ActRIIB-mFc in wild-type as well as Akt1ko and Akt2ko mice. However, ActRIIB-mFc treatment did not improve the distance run or endurance during treadmill exercise. It is unclear whether a longer treatment would reveal distinct effects of Akt deficiency on exercise.

*In vitro* treatment with the ActRIIB ligand, myostatin, has been shown to increase the expression of atrophy-mediating E3-ubiquitin ligases, MAFbx/Atrogin-1 and MuRF1 [21], [34]. Additionally, overexpression of Akt in cultured myotubes inhibits glucocorticoid-induced expression of MAFbx [35]. Consistent with these observations, *in vivo* ActRIIB inhibition increased Akt protein levels and decreased mRNA expression of MAFbx and MuRF1 in gastrocnemius, suggesting inhibition of muscle atrophy.

In summary, our results demonstrate that Akt1 and Akt2 are important regulators of muscle size and function; however, neither Akt1 nor Akt2 isoforms are essential for the response to ActRIIB inhibition. In support of our findings, Sartori et al. found that ActRIIB inhibition in muscles where Akt was constitutively active (c.a.Akt) led to even greater hypertrophy than c.a.Akt alone suggesting that ActRIIB and Akt promote muscle hypertrophy through distinct but connected pathways [10]. We speculate that S6K plays a key role in ActRIIB-mFc induced hypertrophy independent of PI3K/Akt as was the case in the response to mechanical stretch in cultured myotubes [36]. Future studies in muscle-specific Akt-deficient mice will provide insights into potential interactions between Akt and ActRIIB, and the implications for growth and function of muscle.

## Methods

### Immunoblotting

Gastrocnemius muscle was homogenized in lysis buffer containing 50 mM Tris·HCl (pH 7.4), 250 mM mannitol, 50 mM NaF, 1 mM sodium pyrophosphate, 1 mM benzamidine, and 1 mM phenylmethylsulfonyl fluoride with 0.5% (wt/vol) Triton X-100, supplemented with complete protein inhibition cocktail tablet from Roche (Penzberg, Germany) as described previously [37]. Protein extracts (30 µg) were separated by 4–12% NuPAGE Bis-Tris gel (Invitrogen) and transferred to nitrocellulose membranes with semidry transfer cells (Bio-Rad Laboratories, Hercules, CA). After 1 h of blocking with Tris-buffered saline with 0.1% (vol/vol) Tween 20 containing 3% (wt/vol) nonfat dried milk, membranes were incubated with a polyclonal antibody against phosphorylated (Ser473) Akt and Akt1/2/3 (Santa Cruz). Additionally blotted for Akt1, Akt2, phosphorylated (Ser9, Ser21) Glycogen synthase kinase-3, mammalian target of rapamycin, phosphorylated (Thr389) S6 kinase, phosphorylated (Ser240/244) S6 ribosomal protein, S6 ribosomal protein, glyceraldehyde-3-phosphate dehydrogenase, and β-actin (Cell Signaling). The signals were detected with enhanced chemiluminescence (ECL, Amersham), and film autoradiograms were analyzed with laser densitometry and Photoshop CS3 (Adobe).

### Animals and treatment

All animal work was reviewed and approved by the Institutional Animal Care and Use Committee of the University of Pennsylvania School of Medicine (Protocol # 701656). Eight-week-old male wild-type (WT) C57BL/6J mice (Jackson Laboratories, Bar Harbor, ME) and Akt1ko [7] and Akt2ko mice [11] were housed (n = 5 per cage) under a 12: 12-h light-dark cycle (light on at 0700) and an ambient temperature of 22°C, and allowed free access to water and chow diet. ActRIIB-mFc (also termed RAP031, 10 mg/kg) provided by Acceleron Pharmaceuticals, Cambridge, MA) was injected intraperitoneally twice weekly for 10 weeks [15]. The vehicle was phosphate-buffered saline (PBS). Mice were fed regular chow diet (LabDiet, Richmond, IN, # 5001, containing 4.5% fat, 49.9% carbohydrate, 23.4% protein; 4 kcal/g). Food intake was measured weekly, and body composition was assessed prior to treatment and 10 weeks later with nuclear magnetic resonance (NMR) (Echo Medical Systems, Houston, TX) [15], [37].

### Treadmill and grip strength

ActRIIB-mFc or vehicle-treated mice were acclimatized to a modular treadmill connected to an open circuit indirect calorimeter (CLAMS, Columbus Instruments, Columbus, OH). After 10 weeks of treatment, the mice were deprived of food for 5 hours in the morning, and underwent forced exercise on the treadmill (angle 10°, 15 min at 10 meters/min and then 15 meters/min until exhaustion). Work done (J) was calculated using the following equation: mg (µ_k_cosθ+sinθ)*[cosθdx+sinθdy] where m = mass, g = 9.81 m/s^2^, µ_k_ = 0.987, θ = angle of treadmill incline, dx = horizontal distance, dy = vertical distance.

The muscle strength in the forelimbs was measured with a grip meter (TSE; Bad Hamburg, Germany) as previously described [15]. Briefly, mice were trained to grasp a horizontal metal bar while being pulled by their tail and the force was detected by a sensor. Ten measurements were determined for each mouse and averaged.

### EDL contraction

The mice were euthanized with carbon dioxide, muscles were dissected and weighed, and contraction of the extensor digitorum longus (EDL) muscle was analyzed *ex vivo*. Physiological measurements, including contraction time, half-relaxation time, peak isometric twitch force, and peak isometric tetanic force were analyzed in freshly dissected EDL muscles as previously described [38]. Briefly, muscles were stimulated in Ringer's solution composed of 100 NaCl mM, 4.7 KCl mM, 3.4 mM CaCl_2_, 1.2 mM KH_2_PO_4_, 1.2 mM MgSO_4_, 25 mM HEPES, and 5.5 mM D-glucose. Muscle length was adjusted to obtain the maximal twitch response, and this length was measured and recorded as optimal length. Peak twitch and tetanus forces were obtained from three maximal contractions (120-Hz stimulation frequency, 500-ms stimulation duration).

### Histology

At the end of the physiological studies, EDL and soleus muscles were flash-frozen in isopentane cooled in liquid nitrogen and stored at −80°C prior to sectioning. Serial frozen sections (12 µm) were cut at mid-belly of the EDL muscle using a cryostat at −21°C and placed onto glass slides (Superfrost/Plus, Fisher Scientific). Single-fiber cross-sectional area (CSA) and distribution were determined in images from tissue sections immunostained for laminin [39]. Photomicrographs were taken using an Olympus BX51 microscope equipped with a Magnafire camera. Morphometric measurements were made with Photoshop CS3 (Adobe). Fiber data was used to calculate an average single-fiber area and plot it as a histogram. For ease of interpretation, histograms are presented as smoothened approximations by constructing a spline function (GraphPad Prism), whose average value over each bar interval equals the height of that bar. The total number of fibers was counted per muscle section. Type I and type II fibers were distinguished through metachromatic ATPase method [40], [41].

### Glycogen content

Quadriceps muscles were dissected, weighed, and flash frozen in liquid nitrogen. Samples were homogenized in 0.03 N HCl. The homogenate (100 µl) was mixed with 400 µl of 1.25 N HCl and heated for 1 h at 100°C. Samples were centrifuged at 14,000 rpm, and 10 µl of supernatant was mixed with 1 ml of glucose oxidase reagent (Sigma). After a 10-min incubation at 37°C, the absorbance was read at 505 nm. A standard curve using glycogen type III obtained from rabbit liver (Sigma) was also simultaneously analyzed to determine the final liver glycogen concentrations.

### Statistical analysis

The effects of genotype and ActRIIB-mFc treatment on various parameters were analyzed by ANOVA, and pair-wise differences were determined with student's t-test.

## Supporting Information

Figure S1 Effects of Akt deficiency and ActRIIB inhibition on MAFbx and MuRF1 expression. Effects of genotype and ActRIIB-mFc treatment (Rx, black bar) or vehicle (Veh, white bar) in wild-type (WT), Akt1 knockout mice (Akt1ko), and Akt2 knockout mice (Akt2ko) on MAFbx (Fbxo32) and MuRF1 (Trim63) expression. Expression values are normalized to phosphoriboprotein (36B4; Rplp0). Data are mean ± SEM, n = 5. **P<0.01 vs. same genotype treated vehicle.
(0.13 MB TIF)
