# Supplementary figures and images for: Akt Deficiency Attenuates Muscle Size and Function but Not the Response to ActRIIB Inhibition
Source: PLoS One. 2010 Sep 15;5(9):e12707. doi: 10.1371/journal.pone.0012707 (PMC2939888; doi:10.1371/journal.pone.0012707)

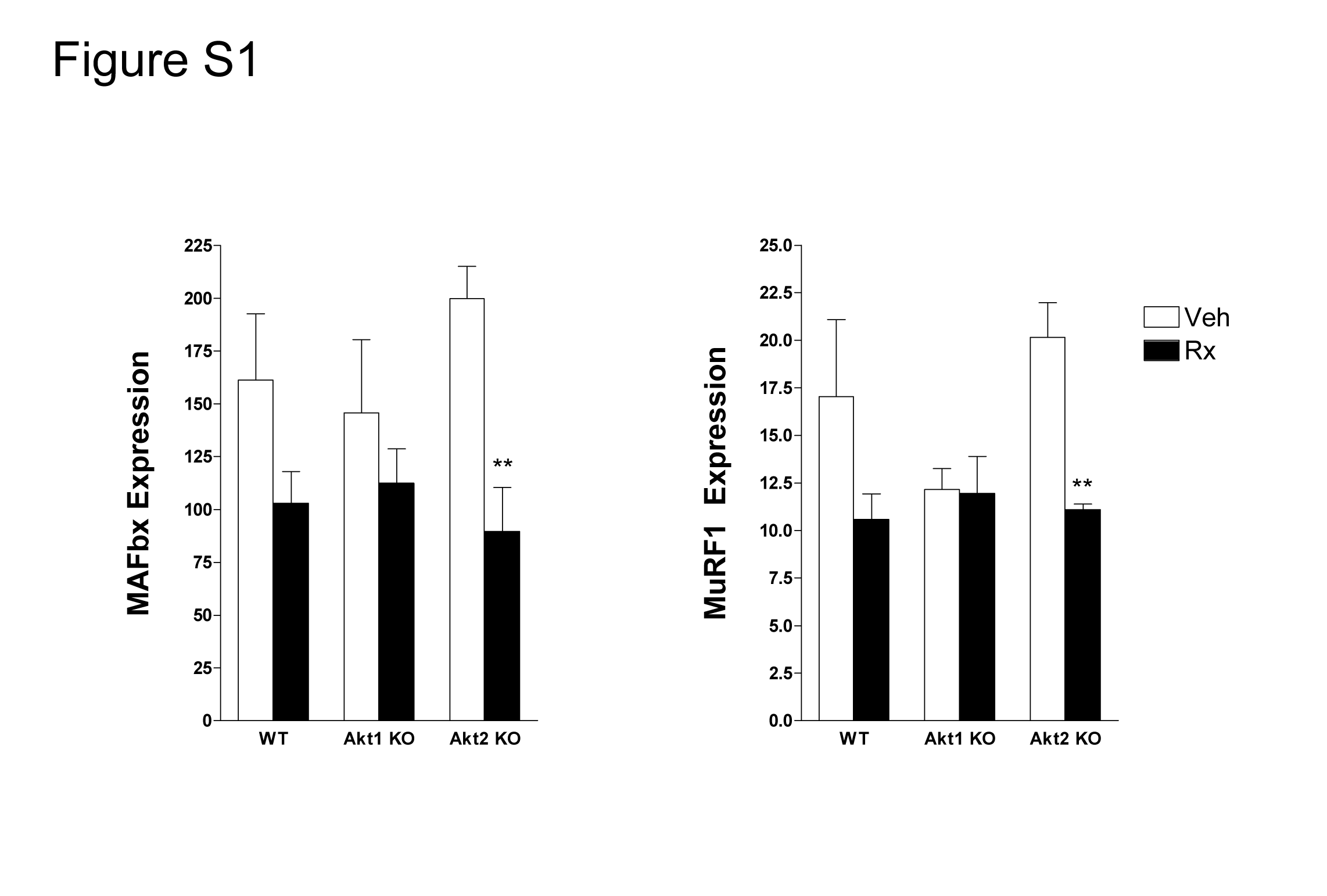

Supplement: Figure S1 — Effects of Akt deficiency and ActRIIB inhibition on MAFbx and MuRF1 expression. Effects of genotype and ActRIIB-mFc treatment (Rx, black bar) or vehicle (Veh, white bar) in wild-type (WT), Akt1 knockout mice (Akt1ko), and Akt2 knockout mice (Akt2ko) on MAFbx (Fbxo32) and MuRF1 (Trim63) expression. Expression values are normalized to phosphoriboprotein (36B4; Rplp0). Data are mean ± SEM, n = 5. **P<0.01 vs. same genotype treated vehicle. (0.13 MB TIF) [file pone.0012707.s001.tif]
